# Oncolytic Viruses: Do They Have a Role in Anti-Cancer Therapy?

**DOI:** 10.4137/cmo.s416

**Published:** 2008-02-09

**Authors:** Robin J Prestwich, Fiona Errington, Kevin J. Harrington, Hardev S. Pandha, Peter Selby, Alan Melcher

**Affiliations:** 1 Cancer Research UK, St James’s University Hospital, Beckett Street, Leeds, LS9 7TF, UK; 2 Targeted Therapy Laboratory, Institute of Cancer Research, Cancer Research UK. Centre for Cell and Molecular Biology, Chester Beatty Laboratories, 237 Fulham Road, London SW3 6JB, UK; 3 Head and Neck Unit, Royal Marsden Hospital, 203 Fulham Road, London SW3 6JJ, UK; 4 Oncology, Postgraduate Medical School, University of Surrey, Guildford, GU2 7XX, UK

**Keywords:** oncolytic virus, tumor selectivity, safety, virus delivery, anti-tumour immunity, administration

## Abstract

Oncolytic viruses are replication competent, tumor selective and lyse cancer cells. Their potential for anti-cancer therapy is based upon the concept that selective intratumoral replication will produce a potent anti-tumor effect and possibly bystander or remote cell killing, whilst minimizing normal tissue toxicity. Viruses may be naturally oncolytic or be engineered for oncolytic activity, and possess a host of different mechanisms to provide tumor selectivity. Clinical use of live replicating viruses is associated with a unique set of safety issues. Clinical experience has so far provided evidence of limited efficacy and a favourable toxicity profile. The interaction with the host immune system is complex. An anti-viral immune response may limit efficacy by rapidly clearing the virus. However, virally-induced cell lysis releases tumor associated antigens in a ‘dangerous’ context, and limited evidence suggests that this can lead to the generation of a specific anti-tumor immune response. Combination therapy with chemotherapy or radiotherapy represents a promising avenue for ongoing translation of oncolytic viruses into clinical practice. Obstacles to therapy include highly effective non-specific host mechanisms to clear virus following systemic delivery, immune-mediated clearance, and intratumoral barriers limiting virus spread. A number of novel strategies are now under investigation to overcome these barriers. This review provides an overview of the potential role of oncolytic viruses, highlighting recent progress towards developing effective therapy and asks if they are a realistic therapeutic option at this stage.

## Introduction

The anti-tumor activity of viruses has been observed throughout the 20th century. Viral infections were noted to precede apparently inexplicable cancer remissions in multiple case reports of spontaneous remissions (Bluming and Ziegler, 1971; DePace, 1912; Hansen and Libnoch, 1978). Viruses were tested as anti-cancer agents in the 1950’s, before research into this field was widely abandoned due to a lack of efficacy and toxicity concerns (Kelly and Russell, 2007). More recent scientific developments in the fields of virology, genetic manipulation, molecular and cell biology have provided insights into the mechanisms by which viruses may selectively infect cancer cells, and of how viruses may be manipulated to enhance their anti-cancer activity. In 1991, the anti-tumor activity of herpes simplex virus was demonstrated in a murine glioma model (Martuza et al. 1991), and was followed by a resurgence of interest in the use of viruses as novel cancer therapy. A host of viruses have subsequently been investigated for their oncolytic activity, and several have entered clinical trials over the last 10 years (Aghi and Martuza, 2005).

Oncolytic viruses are self-replicating, tumor selective and lyse cancer cells following viral infection. Non-oncolytic non-replicating viruses, in contrast, may also be used in cancer therapy, but as vectors to deliver gene therapy. Oncolytic viruses are dependant upon the host for replication, and differences in the cellular processes of normal host cells and tumor cells provide the potential for tumor-selective replication. Characteristics of cell transformation, including increased cell cycling, oncogene activation, altered receptor expression and defective signalling pathways, have been shown to enhance the ability of some viruses to replicate in neoplastic cells (Stojdl et al. 2000; Barber, 2005; Shmulevitz et al. 2005). Oncolytic viruses may be tumor selective in their wild type or attenuated forms, or may be genetically modified to provide or enhance tumor selectivity (Parato et al. 2005). The potential utility of oncolytic viruses as anti-cancer therapy is based upon the concept that replication of the virus within the tumor will amplify viral load and enhance anti-tumor potency, whilst tumor selectivity will provide a favourable toxicity profile, sparing normal tissues. Non-overlapping mechanisms of action and toxicity profiles will allow combination with standard therapies. In addition, although the anti-tumor activity of oncolytic viruses is conventionally considered to be mediated via the direct actions of the virus upon tumor cells, there is emerging evidence that tumor infection can lead to the generation of an effective anti-tumor immune response (Diaz et al. 2007; Moehler et al. 2005; Greiner et al. 2006; Schirrmacher, 2005).

Significant obstacles need to be overcome in the design of effective oncolytic virus treatment schedules. Locally administered virus does not generally spread to other tumor sites (Liu and Kirn, 2007), and systemically administered virus is highly susceptible to efficient immune and non-immune mediated clearance mechanisms (Fisher, 2006). Even if tumor infection is successfully achieved, physical barriers within the tumor environment prevent efficient infection throughout the tumor. Modification of viruses to provide tumor selectivity and to minimise toxicity may reduce anti-tumor potency (Gunzburg, 2005). In addition, significant safety concerns exist regarding the use of replication competent viruses (Chernajovsky et al. 2006). Viruses do not adhere to conventional toxicity and dose-response relationships, and optimum dose schedules remain unresolved (Aghi and Martuza, 2005).

This review aims to provide an introduction to the potential of oncolytic viral therapy, highlighting progress in understanding critical interactions with the immune system and the recent development of strategies designed to overcome current obstacles to successful therapy.

## Naturally occurring oncolytic viruses

A group of naturally occurring or attenuated replication competent oncolytic viruses have been identified, which are non-pathogenic or induce only mild symptoms in humans, and possess intrinsic tumor selectivity (Roberts et al. 2006). This group includes reovirus, a human virus with low pathogenicity (TylerKL, 2001), and viruses including Newcastle disease virus (NDV) (Schirrmacher, 2005), and vesicular stomatitis virus (Diaz et al. 2007), for which humans are not normally hosts. In order to develop vaccines, pathogenic human viruses have been attenuated by repeated passage in tissue culture. Some of these strains, including measles (Grote et al. 2001), have acquired the property of oncolytic restriction during this process. The use of naturally occurring as opposed to genetically modified viruses is advantageous in human therapy in terms of regulatory restrictions and safety concerns (Chernajovsky et al. 2006).

## Genetically Modified Oncolytic Viruses

Viruses use a range of strategies to regulate host cellular processes and promote efficient replication within host cells. Genetic modification of viruses can be used to interfere with these viral strategies, preventing replication within normal host cells, whilst permitting replication in tumor cells. Replacement or modification of viral promoters can provide tumor selectivity (Ko et al. 2005). Human DNA viruses, including adenovirus (McCormick, 2003), herpes simplex virus (Martuza et al. 1991) and vaccinia virus (Zeh and Bartlett, 2002), have been modified using recombinant technology. This group of DNA viruses are additionally modified to attenuate their pathogenicity. In addition to safety concerns regarding the use of genetically modified viruses, modification to reduce pathogenicity can also attenuate anti-tumor potency (Gunzburg, 2005).

## Mechanisms of Tumor Specificity

The process of carcinogenesis involves genetic instability, with one estimate of over 11,000 genomic alterations occurring in a cancer cell (Stoler et al. 1999). Neoplastic cells are selected for growth advantage, but these mutations can lead to defects in anti-viral defences, altered expression of receptors, expression of novel receptors, and disrupted intracellular signalling pathways (Vaha-Koskela et al. 2007). In view of these diverse changes, it is perhaps not surprising that the tumor specificity of oncolytic viruses can be mediated by an array of different mechanisms, and that in many cases the basis of oncolytic restriction has been incompletely elucidated (Parato et al. 2005). A whole range of mechanisms have been exploited to engineer or enhance tumor specificity (McCormick, 2001). Mechanisms of tumor selectivity can be broadly catagorised into five groups: defective anti-viral defences which are particularly exploited by naturally oncolytic viruses, targeting to receptors unique to or over-expressed on tumors, the use of tumor or tissue specific promoters, viral gene deletions or mutation restricting viral replication in normal tissue, and proteolytic processing of viruses in the tumor microenvironment.

### Defective anti-viral responses

Anti-viral defence mechanisms are inactived in many tumors. The double stranded RNA (dsRNA) dependant protein kinase (PKR) and type I interferon (IFN) pathways are important, overlapping, anti-viral mechanisms in normal cells.

Reovirus is an example of a naturally occurring oncolytic virus, whose tumor selectivity involves a defective PKR anti-viral response. Reovirus has been found to replicate more rapidly in Ras-activated cells (Shmulevitz et al. 2005; Coffey et al. 1998). In normal cells viral dsRNA induces activation and phosphorylation of PKR, which in turn inhibits translation of viral transcripts via phosphorylation of transcription initiation factor 2α (eIF2α) (de Haro et al. 1996). In susceptible Ras-transformed cells, PKR is not phosphorylated in response to reovirus infection (Strong et al. 1998). In common with other oncolytic viruses, the mechanism of oncolytic restriction is complex, with recent studies showing that Ras-transformation enhances viral uncoating, infectivity and virion release, in addition to an effect on the function of PKR (Marcato et al. 2007).

The type I interferons, IFNα and IFNβ, have anti-proliferative properties, and defects in these pathways are commonly found in tumors, promoting cancer growth (Roberts et al. 2006). Several oncolytic viruses exploit defects in the IFN pathways (Stojdl et al. 2000). For example, NDV is able to inhibit IFN signalling in avian but not human cells, and is therefore not a human pathogen. NDV can however infect and lyse human tumor cells lacking an intact type I IFN response (Schirrmacher, 2005).

### Receptor targeting for tumor selective uptake

Viruses can achieve tumor specificity if they obtain entry to cells via receptors that are overexpressed on tumor cells or via mutated receptors that are unique to tumor cells. For example, the Edmonton strain of measles virus can obtain entry to cells via CD46, a receptor which is overexpressed in some tumors (Fishelson et al. 2003). Viruses can also be retargeted to tumors by modifying virus attachment proteins. The measles haemagglutinin (H) attachment protein has been altered to act as a ligand for receptors, including growth factor receptors, found on tumors (Nakamura et al. 2005). This approach relies upon the identification of tumor-selective receptor targets, and ensuring that normal tissues are not infected by the modified virus.

### Targeting to tumor or tissue specific promoters

Viruses can be genetically modified to provide tumor selectivity by placing regulatory viral genes under control of a tumor or tissue specific promoter. Promoters for tumor antigens such as prostate specific antigen and alphafetoprotein have been incorporated into genetically modified adenoviruses (Hallenbeck et al. 1999; Ko et al. 2005; Rodriguez et al. 1997). This approach is, however, limited by the weak expression or absence of identifiable tumor or tissue specific promoters in most cancers.

### Genetically engineered virus defects permitting replication in tumor cells

Viral genes can be mutated or deleted to provide selective replication in tumor cells with abnormal cellular pathways. Mutations can allow replication in tumor tissue, in contrast to normal tissue, if the deleted or mutated viral function can be replaced by an altered tumor function. The classic example of a deletion providing tumor selectivity is of a modified adenovirus, ONYX-015 (McCormick, 2003). ONYX-015 was one of the first modified viruses to enter clinical trials, and is engineered to lack the E1B protein. E1B inhibits p53 function, preventing anti-viral responses coordinated by p53 including loss of cell cycling and increased apoptosis. ONYX-015 was designed with the concept that replication would occur selectively in tumors lacking p53, but not in normal cells with intact p53 mechanisms. The mechanism of tumor selectivity now appears more complex, with evidence that E1B has a function in the export of viral RNA. Tumor cells but not normal cells appear to be able to provide the missing export function of E1B (O’Shea et al. 2004).

Vaccinia virus is another example of a deletion-modified virus, with deletion of the viral gene thymidine kinase causing loss of the ability to synthesise nucleotides. The modified vaccinia virus can only replicate in cycling cells, such as transformed cells, with an abundant supply of nucleotides (Zeh and Bartlett, 2002).

### Proteolytic processing of virus particles in the tumor microenvironment

Proteolytic disassembly of the outer viral coat is required for successful infection by several viruses, including reovirus, NDV and adenovirus (Alain et al. 2007; Medina-Kauwe, 2003). For example, the action of proteases converts reovirus into the infectious intermediate sub-viral particles (ISVP). Reovirus ISVPs are able to successfully infect cell lines which are resistant to intact reovirus (Alain et al. 2007). The tumor microenvironment is commonly associated with the presence of elevated concentrations of proteases, which play a role in tumor invasion and metastasis (Mohamed and Sloane, 2006). This proteolytic tumor microenvironment provides the opportunity to enhance the tumor selectivity, by modifying the viruses to be dependant upon the action of proteases for optimal infectivity. For example the fusion (F) protein of measles virus, which facilitates entry into cells, has been modified such that it requires the action of matrix metalloproteinase 2 for conversion into the functional form (Maisner et al. 2000).

## Animal Models

Animal models are widely used prior to new therapies entering human clinical trials, although their limitations are well recognised (Mestas and Hughes, 2004). The use of animal systems when investigating oncolytic viruses is particularly problematic in view of species variability in the efficiency of infection and pathogenicity (Vaha-Koskela et al. 2007). This has led to the use of immunocompromised human xenograft models, which inevitably are unable to accurately predict the interaction between virotherapy and an immunocompetant host. Consequently animal models can only provide a limited insight regarding efficacy and toxicity of human oncolytic virotherapy.

## Clinical Experience with Oncolytic Viruses

The experience from clinical trials using oncolytic viruses has been summarised by Aghi et al. (Aghi and Martuza, 2005). For use in clinical studies manufacture of the virus according to the principles of Good Manufacturing Practices to a high titre must be possible. Several different oncolytic viruses have entered modern clinical trials since 1996, including naturally oncolytic reovirus, NDV and Coxsackievirus, attenuated and modified measles virus, and engineered adenovirus, vaccinia, and HSV. Administration routes have included intratumoral injection, systemic and intracavitary delivery. Overall clinical data suggests a limited degree of efficacy with several oncolytic viral therapies. For example, ONYX-015 has entered a series of clinical trials, with local tumor regression rates of between 0–14% (Kirn, 2001). This level of anti-tumor activity, although low, provides encouragement for future oncolytic viral therapy if the route of administration, potency, tumor selectivity and immune interactions can be optimised. In contrast to conventional phase I drug trials, the maximum tolerated dose of virus is commonly not reached, and dose is limited by technical restrictions in the quantity of virus which can be produced (Parato et al. 2005). Replication competent viruses do not have a straightforward dose response relationship for efficacy or toxicity, and the optimum dosing regimens for most viruses remain unclear. If the virus is able to rapidly replicate in a tumor, it may only be necessary for a low viral load to infect tumor tissue. Anti-viral humoral immune responses may confound dose effects. Evidence of a dose response exists for the use of systemically administered NDV, although not for some other viruses including HSV (Aghi and Martuza, 2005).

The majority of clinical studies have administered virus via intratumoral injections (Liu and Kirn, 2007). Mechanisms which provide viruses with tumor selectivity do not completely preclude infection of normal tissue and consequent toxicity. Intratumoral delivery of oncolytic viruses provides a further direct physical restriction to enhance tumor selectivity. Considerable safety data has been obtained from these studies, and local delivery of viral therapy is generally very well tolerated, with the most common side effects being mild ‘flu-like symptoms and a minor local reaction (Kirn, 2001). There has been evidence of clinical activity in some of these early clinical studies, ranging through stable disease, marker responses, to partial and occasional complete responses (Aghi and Martuza, 2005). Studies with intratumoral administration have generally not demonstrated activity against distant non-injected lesions (Liu and Kirn, 2007), greatly limiting the potential of local viral therapy in the treatment of metastatic disease. In an exception to this observation, in patients with metastatic melanoma receiving intratumoral injections of GM-CSF armed vaccinia virus (JX-594, JENNEREX), regressions in distant non-injected dermal metastases were noted in four of seven patients treated (Mastrangelo et al. 1999). These regressing lesions were found to be heavily infiltrated by T lymphocytes. Similarly, in a phase I study of intratumoral administration of a modified HSV expressing GM-CSF, non-injected distant tumor sites became inflamed in 4 of 30 patients (Hu et al. 2006).

A limited number of studies have investigated intravenous delivery of virus. Studies using intravenous administration of the modified adenoviruses have not shown systemic activity (Liu and Kirn, 2007). Systemic efficacy following intravenous administration has however been demonstrated using NDV (Lorence et al. 2003) and reovirus (Stoeckel and Hay, 2006; Spicer et al. 2007). In a phase I study of intravenously administered reovirus in patients with advanced cancer, viral replication was demonstrated in post-treatment tumor biopsies and stable disease was reported in 6 of 32 evaluable patients (Spicer et al. 2007), demonstrating the feasibility of systemic viral delivery to tumors. Systemic delivery is associated with more severe ‘flu-like symptoms, although the toxicity profile remains favourable compared with conventional cancer therapy. Dose schedule influences toxicity, with a prolonged infusion time reducing toxicity following systemic delivery of NDV (Hotte et al. 2007). Observations with dose schedules with one virus are not necessarily applicable to other viruses. One treatment-related death has been reported, in a patient with reduced lung capacity, following treatment with a naturally modified NDV. Post-mortem examination suggested that rapid tumor lysis had led to respiratory failure (Pecora et al. 2002). Similar to conventional cancer therapy, this case demonstrates the need for adequate performance status and functional reserve.

## Clinical Lessons Learnt from Important Oncolytic Viruses

### Onyx-015

One of the most studied oncolytic viruses to date is Onyx-015. This E1B gene deleted adenovirus was the first oncolytic viral agent to be tested in humans and over 200 cancer patients have so far been treated in over 15 clinical trials (phases I–III)(Kirn, 2001). It was developed on the hypothesis that an adenovirus with a deletion in the p53-inhibitory gene E1B would replicate in tumours in which p53 was defective (Bischoff et al. 1996). As it was the first agent of its kind to be tested in humans it was deemed important to assess biological data on a viral replication and antiviral immune responses in the initial clinical trials (Kirn, 2001). A staged approach to clinical development was adopted in which the virus was first tested intratumorally to assess it safety and biological activity and then subsequent trials studied administration via intraperitoneal, intra-arterial and eventually intravenous administration. The virus was well-tolerated at the highest doses that could be administered (2 × 10^12^– 2 × 10^13^ viral particles), within the limits of viral manufacture, by all routes. Flu-like symptoms were the most common toxicities and were increased in patients receiving vascular administration. There was no clear correlation between the development of flu-like symptoms and viral dose or dose administration regimen. Neutralizing antibodies were generated following all routes of administration and viral doses. Interestingly, following intra-peritoneal delivery to patients with ovarian cancer, toxicity including viraemic symptoms and abdominal pain appeared more commonly in patients with bulky disease (Vasey et al. 2002). As the access of intraperitoneally administered drugs into solid tumour nodules is limited and retention of the virus within the abdominal cavity was generally of short duration, it was postulated that intraperitoneal viral therapy is most likely to efficacious in patients with low tumour burden (Vasey et al. 2002). In these patients the severity of flu-like symptoms was unrelated to the presence or absence of pre-exisiting neutralizing antibodies. Antitumoral activity was demonstrated using single agent Onyx-015 intratumoral therapy in head and neck cancer, although the response rate was only 13% following repeated treatment (Nemunaitis et al. 2000; Kirn, 2001). These responses did not correlate with neutralizing antibody levels, potentially due to the inefficient penetration of antibodies into tumor masses (Baxter et al. 1994). No objective responses with single agent therapy occurred in patients with pancreatic, colorectal or ovarian cancers. In view of the rarity of clinical responses to Onyx-015 combination therapy with chemotherapy was explored. As discussed later, a synergistic interaction with chemotherapy in multiple tumours types and by multiple routes of administration was observed (Heise et al. 1997; Kirn, 2001).

### HSV1716

HSV1716 is an engineered oncolytic virus which lacks copies of the gene encoding the virulence factor ICP34.5 (Dolan et al. 1992) such that it will only replicate in actively dividing cells and not terminally differentiated cells (Brown et al. 1994). HSV1716 has been shown to be safe when administered intratumorally in patients with recurrent high-grade glioma (HGG) (Rampling et al. 2000) and metastatic melanoma (MacKie et al. 2001). In both tumours the virus has been shown to replicate without causing toxicity in both HSV seropositive and seronegative patients (MacKie et al. 2001; Papanastassiou et al. 2002). Prior exposure to HSV does not therefore preclude therapy with HSV1716. Studies in patients with HGG have gone on to explore combination with surgical debulking, with HSV1716 injected into the rim of the surgical resection cavity, followed by chemotherapy and/or radiotherapy as clinically indicated (Harrow et al. 2004). Again no toxicity was evident, and there were encouraging suggestions of prolonged survival in some patients with this poor prognosis tumour. The authors have postulated that the inflammatory environment post-surgery may limit viral replication, and that virus delivery optimisation remains critical to the future success of this therapeutic approach (Harrow et al. 2004). As with Onyx-015, the experience with HSV1716 is suggestive of the potential therapeutic benefit of combined modality treatment.

## Safety

Despite the clinical experience of a favourable toxicity profile with high viral doses, compared with the toxicity of conventional cytotoxic therapies, safety concerns remain concerning the use of replicating oncolytic viral therapy. In contrast to gene therapy with non-replicating adenovirus or retrovirus vectors, the interactions of replicating virus with both host and environment are far more difficult to predict. Live replicating virus can be shed from patients following treatment, raising the possibility of person-to-person transmission. For example NDV has been isolated from urine up to three weeks after treatment (Pecora et al. 2002). The mutation rate of viruses is high, more so in the case of RNA viruses (Chernajovsky et al. 2006). Even if the administered virus does not represent a human pathogen, there is the theoretical possibility that the virus may mutate to acquire pathogenic properties and successfully infect normal host tissues or bystanders. Modified viruses may revert to wild-type, and additionally may recombine with wild-type viruses (Chernajovsky et al. 2006). In order to avoid such scenarios, viruses have been attenuated by mutations and deletions. Unfortunately, attenuated viruses may also lose anti-tumor potency (Gunzburg, 2005). Pro-drug activating suicide genes have been incoporated into some modified viruses as a safety mechanism in order to eliminate infected cells in case infection runs out of control (Gunzburg, 2005). Therapeutic strategies to improve oncolytic virotherapy include enabling the virus to evade immune-mediated clearance, potentially circumventing the immune barrier to unwanted viral spread. It is important that consideration of these safety issues continues to influence the development of oncolytic viral therapy.

## The Potential of Oncolytic Viruses to Induce Anti-Tumor Immunity

Despite intensive investigation of the direct cytotoxic ability of oncolytic viruses, little attention has been given to the potential to induce anti-tumor immunity. Matzinger has proposed that the key role of the immune system is not primarily the identification of self and non-self, but the recognition of ‘danger’ signals (Matzinger, 1994). Virus-induced cell death is expected to create an inflammatory ‘dangerous’ environment, with pro-inflammatory cytokine release, the presence of toll-like receptor (TLR) ligands and infiltration of cells of the innate immune system (Matzinger, 1998; Zeng et al. 2002; Alexopoulou et al. 2001). The immune response to a foreign challenge involves both the innate and acquired arms of the immune system, with the innate immune response guiding subsequent antigen specific adaptive immunity (Gallucci and Matzinger, 2001). In addition to this ‘dangerous’ context, virally-induced lysis of tumor cells will release a wide range of tumor associated antigens (TAA) into the microenvironment. Infiltrating dendritic cells can process released TAA, migrate to lymph nodes, cross-present antigen to T cells, and potentially generate an adaptive anti-tumor immune response (Toda et al. 1999).

The inflammatory context created by virus-induced cell death is more likely to produce a stimulatory immune response as opposed to tolerance (Schirrmacher, 2005). However, due to the immunogenicity of viral antigens, an anti-viral adaptive immune response may rapidly clear virus and inhibit the development of anti-tumor immunity. In addition, the ability to prime an effective anti-tumor adaptive immune response is dependant upon the ability of dendritic cells to cross present TAA in an appropriate costimulatory context to T cells (Banchereau et al. 2001), and many viruses are recognised to interfere with DC function as part of their immune evasion mechanisms (Pollara et al. 2005).

There is some limited data regarding the ability of oncolytic viruses to generate an anti-tumor immune response, drawn from animal models, *in vitro* human systems and clinical trials.

### Animal models

Different modified strains of HSV have been shown to induce systemic anti-tumor immune response in murine models (Toda et al. 1999; Li et al. 2007a; Li et al. 2007b). In one of these studies, in mice with established bilateral colorectal or melanoma tumors, unilateral intratumoral injection of an attenuated strain of HSV elicited a reduction in the size of the contralateral uninjected tumor, associated with generation of CD8 T cells specific to a tumor antigen (Toda et al. 1999). Diaz et al. (Diaz et al. 2007) used VSV in a B16ova murine melanoma model, finding in cellular depletion studies that intact CD8+ T cells and natural killer cells are critical to the efficacy of intratumoral VSV therapy. CD8+ cells were detected both to viral epitopes and to the SIINFEKL epitope of the model tumor antigen, OVA. In a tumor vaccination rat model, an oncolytic virus parvovirus H-1 was found to enhance the ability of lethally irradiated autologous tumor cells to suppress tumor growth (Raykov et al. 2007).

### In vitro human systems

Moehler et al. (Moehler et al. 2005) used an *in vitro* allogeneic human system to provide ‘proof of principle’ that oncolytic virus-induced cell death can lead to cross presentation of TAAs. Tumor cell lysates induced by parvovirus H-1 stimulated DC maturation and cross presentation of melanoma antigens to CTL clones, in contrast to tumor cell lysates induced by ultraviolet light or freeze-thaw cycles. Greiner et al. (Greiner et al. 2006) investigated the ability of a highly attenuated modified vaccinia virus to prime an adaptive anti-tumor response. Dendritic cells cocultured with an allogeneic melanoma cell line infected by the modified virus were able to induce a mixed leukocyte response, and to prime autologous T cells to generate an interferon response to a melanoma TAA, MelanA. In contrast, an unattenuated wild type strain of the vaccinia virus, Western Reserve (WR) inhibited DC maturation (Jenne et al. 2000) and was not capable of priming an immune response.

### Clinical strategies

Oncolytic viruses have been incorporated into different strategies with the aim of generating anti-tumor immunity.

#### Ex vivo oncolysates

Oncolytic viruses have been incorporated into tumor vaccine preparations in an attempt to enhance their immunogenicity. Phase II trials investigating the use of vaccinia virus melanoma cell lysates in the adjuvant setting following resection of melanoma suggested highly significant survival improvements compared with historical melanoma patient controls (Hersey et al. 1987). Disappointingly a subsequent phase III study of 700 patients failed to show any improvement in recurrence or overall survival (Hersey et al. 2002).

Schirrmacher et al. (Schirrmacher, 2005) have performed a series of clinical vaccine studies using a live autologous tumor vaccine, infected by NDV, followed by high dose irradiation to render tumor cells non-viable. Using skin prick tests following NDV autologous tumor vaccinations, a significant number of patients developed a specific anti-tumor delayed type hypersensitivity memory response. A variety of phase II studies have subsequently been performed to evaluate clinical efficacy of this vaccination approach. Studies in colorectal cancer (Schlag et al. 1992), glioblastoma multiforme (Steiner et al. 2004), malignant melanoma (Schirrmacher et al. 1998), and breast cancer (Schirrmacher, 2005) have demonstrated statistically significant improvements in overall survival by 20–36% at 2–5 year follow up. These observed improvements in survival lend support to the theory that the danger signal provided by oncolytic viruses may break tumor immune tolerance, but as demonstrated by the experience with vaccinia virus, prospective phase III trials are required.

#### In situ tumor injection

In clinical studies, the previously discussed regression of non-injected distant melanoma deposits with associated lymphocyte infiltrate following intratumoral administration of the JX594 modified vaccinia virus suggests the possible development of anti-tumor immunity (Mastrangelo et al. 1999).

Other investigators have started to extend vaccine strategies by arming viral vectors with cytokines and immune costimulatory molecules (Mastrangelo and Lattime, 2002). Successful transfection into tumors has been achieved, although with no convincing clinical benefit to date.

#### Vaccination approaches using viruses transduced with TAA

TAAs have been transduced into viruses in order to enhance the prospect of generating anti-tumor immunity as opposed to anti-viral immunity. In a phase I/II study of non-replicating vaccinia virus expressing melanoma antigens along with costimulatory molecules, melanoma antigen specific lymphocytes were expanded (Zajac et al. 2003).

## Combination Therapy

Combination therapy utilising virotherapy may be beneficial if the treatments used lack cross-resistance, have non-overlapping toxicity or demonstrate synergistic effects on tumor kill. The action of radiotherapy or chemotherapy in combination with virotherapy is likely to be complex, with interactions occurring at a cellular level, upon tumor structure and intratumoral barriers to viral spread, and immune modulatory/suppressive effects.

### Radiotherapy

Intratumoral virotherapy can be combined with radiotherapy with the aim of enhancing locoregional control. In preclinical studies there is an increasing body of evidence that radiotherapy can synergise with viral oncolytic therapy (Advani et al. 2006). For example, radiotherapy and modified HSV variants demonstrate supra-additive cell kill in *in vitro* assays of human cervical cancer (Blank et al. 2002) and colorectal cancer (Stanziale et al. 2002). Cellular changes induced by irradiation can enhance the ability of viruses to replicate and spread within a tumor. In a human glioma xenograft model irradiation combined with a modified HSV produced greater tumor reduction than either treatment alone, and an increased viral load was recovered in irradiated grafts (Advani et al. 1998). Similar interactions have been reported between oncolytic adenovirus vectors and radiotherapy. Combined therapy with ONYX-015 and radiotherapy increased tumor growth delay compared with either treatment alone in a colorectal xenograft model (Rogulski et al. 2000). A completed phase I study combining intratumoral injections of reovirus with radiation has demonstrated the feasibility of combination virotherapy with fractionated radiotherapy regimens, and phase II studies are currently underway (Harris et al. 2007).

### Chemotherapy

Synergy has been demonstrated between modified adenoviruses and chemotherapeutic agents (Kirn, 2001). Anti-tumor efficacy was greater in a human xenograft tumor model following administration of ONYX-015 with cisplatin or 5-fluorouracil than with either agent alone (Heise et al. 1997). Based upon encouraging pre-clinical and phase I and II data, a modified ONYX-015 adenovirus vector has been combined with cisplatin and 5-fluorouracil chemotherapy in the treatment of squamous cell carcinoma of the head and neck or oesophagus in a phase III trial. The response rate with viral combination therapy of 79% was significantly greater than that with chemotherapy alone of 40% (Xia et al. 2004).

## Obstacles and Solutions to the Design of Successful Oncolytic Viral Therapy

Regardless of the mechanisms of tumor selectivity possessed by a virus, therapeutic quantities of virus must enter the tumor, spread throughout the tumor, and evade immune-mediated viral clearance long enough to mediate a useful anti-tumor effect. In view of the inability of intratumorally delivered virus to spread to other tumor sites, effective systemic delivery remains a critical goal for the treatment of metastatic disease.

### Virus delivery

The natural host infection route of many viruses involves entry via the skin or mucosa, and viruses are often not adept at survival in the circulation. Evading a series of host defences which remove virus from the circulation is a major challenge for achieving successful systemic administration.

Viruses within the bloodstream are subject to non-specific interactions with blood cells, non-target tissue, the reticulo-endothelial system and complement. Within the blood stream virus particles interact with and bind to circulating blood cells. Binding to blood cells may impair delivery of virus to target tissue. In one experimental system, the binding of an adenovirus to human blood cells compromised delivery to tumor (Lyons et al. 2006). In contrast, binding of virus to blood cells may shield the virus from other mechanisms of clearance, increasing survival in the circulation and allowing for increased interactions with the tumor microcirculation (Cole et al. 2005). Virus particles also adhere to non-blood cells with which they come into contact, removing them from the blood (Pizzato et al. 1999). Circulating virus particles are vulnerable to uptake by the reticuloendothelial system (Ye et al. 2000; Worgall et al. 1997). The liver, in particular, has multiple mechanisms by which virus particles are cleared (Fisher, 2006). The specialised macrophages within the liver, known as Kupffer cells, are likely to play a predominant role in virus removal via scavenger receptors (Worgall et al. 1997). Blood flow through the liver is high, and efficient removal of virus particles by the liver will result in a very short survival in the circulation (Ye et al. 2000). The alternative complement pathway additionally contributes to virus neutralisation in the plasma (Devaux et al. 2004; Wakimoto et al. 2002).

In addition to non-specific interactions which mediate viral clearance, virus particles are cleared from the circulation by antibody interactions. Anti-viral antibodies are generated by the adaptive humoral immune response to circulating virus (Parato et al. 2005), and may neutralise virus particles by binding to the surface of the virus and preventing viral binding to receptors mediating cellular entry (Tsai et al. 2004; Chen et al. 2000). Non-neutralising antibody binding to virus particles enhances virus targeting by complement and uptake by cells bearing Fc receptors (Fisher, 2006). Anti-viral antibodies may pre-exist due to prior viral exposure, and represent a major hurdle to systemic virotherapy. For example, more than 50% of individuals have prior exposure to reovirus infection as demonstrated by the presence of anti-reovirus antibody (TylerKL, 2001). Almost all individuals have circulating anti-measles antibody due to prior vaccination or infection which rapidly neutralises measles virus (Iankov et al. 2007). In addition, an adaptive immune response following treatment generates an antibody response impairing subsequent virus delivery.

Although much initial research focussed upon the tumor selectivity of oncolytic viruses, it is now becoming increasingly clear that these host mechanisms, by which virus is cleared from the circulation, can prevent significant viral infection of tumor targets. A host of potential solutions, in addition to route of delivery, are under investigation to enhance virus delivery.

#### Delivery using cell carriers

The use of infected cell carriers represents a novel strategy to allow virus delivery, ‘shielding’ virus from neutralising antibody (Thorne et al. 2006; Power et al. 2007; Ong et al. 2007; Raykov et al. 2004). Virus may be released into the tumor in response to features of the tumor environment (Harrington et al. 2002; Cole et al. 2005). *In vivo* models have demonstrated the ability of infected monocytic, endothelial, stimulated peripheral blood cells and T cells to deliver measles virus to tumors, in the presence of neutralising antibodies, by a process of cell-to-cell heterofusion induced by the measles virus (Iankov et al. 2007; Ong et al. 2007). Similarly, leukaemic, tumor-derived and xenogenic cell carriers infected with VSV efficiently delivered virus to tumors in model systems, despite the presence of neutralising antibody (Power et al. 2007). Prior to administration in humans these potentially oncogenic cell carriers would need to be inactivated. Thorne et al. (Thorne et al. 2006) utilised cytokine induced killer cells (CIK) as cell carriers for oncolytic vaccinia virus in a murine model. CIK cells are immune cells induced by cytokines *ex vivo*, with anti-tumor activity and an ability to traffic to tumor sites. This dual approach aims to exploit a synergy between virotherapy and cellular therapy. Compared with non-cellular methods of virus delivery, the use of live carrier cells has the potential advantage of viral replication within the carrier cell, leading to the release of an amplified quantity of virus within the tumor environment (Power et al. 2007). Retroviral particles have been found to non-specifically adhere or ‘hitch-hike’ on the surface of T cells, allowing cellular delivery to the tumor in immunocompetant murine models (Cole et al. 2005). The viral particles are released in the tumor microenvironment, in a process enhanced by T cell activation and by heparinase produced by tumor cells. A similar phenomenon has been observed with other viruses (Blomer et al. 2005; Geijtenbeek et al. 2000). If oncolytic viruses can be similarly ‘hitch-hiked’, future potential therapy may combine adoptive T cell transfer with virotherapy.

#### Modification of virus particles to evade clearance

The surface of virus particles, in particular adenovirus, have been modified physically and chemically to mask the viral protein coat, enabling evasion of host clearance mechanisms (Fisher, 2006). These methods are based upon similar strategies designed to reduce drug elimination, although application to virus particles is technically challenging (Fisher, 2006). Examples include encapsulation in a polymer coating (Green et al. 2004; Fisher et al. 2001), microspheres (Matthews et al. 1999) and polyethylene glycol (PEG) (Croyle et al. 2002). Modification can abrogate the ability of viruses to bind to their cellular targets, although ligands to enhance targeting can be incorporated into the encapsulation (Stevenson et al. 2007). In other cases modification does not prevent target infection (O’Riordan et al. 1999).

#### Serotype switching

The anti-viral humoral immune response can prevent effective repeat adminstrations of an oncolytic virus. For some viruses, including VSV and adenovirus, multiple serotypes exist for which neutralising antibodies do not have cross reactivity (Russell, 2002; Bangari and Mittal, 2006). Different serotypes can be sequentially administered, in a process termed serotype switching, to prevent antibody neutralisation.

#### Immunosuppression

Immunosuppressive drugs can be used to inhibit the development of antiviral immunity and early viral elimination. In a murine study of intratumoral reo-virus therapy for colorectal liver metastases the therapeutic efficacy of reovirus was enhanced by immunosuppression by cyclosporin A (Smakman et al. 2006). Another immunosuppressive agent, cyclophosphamide, has been shown to enhance HSV oncolytic therapy in a glioma model, by inhibiting infiltration by phagocytic cells (Fulci et al. 2006). The immune system has complex effects upon viral therapy, and immunosuppression would be expected to reduce the possibility of generating anti-tumor immunity following oncolytic virotherapy. The impact of immunosuppression is likely to be virus and tumor model specific.

#### Inhibition of liver uptake

A novel strategy to enhance the duration of virus circulating in the blood is to inhibit liver uptake (Fisher, 2006). Agents cytotoxic to the Kupffer cells of the liver, such as liposomes containing clodronate, can temporarily inhibit viral uptake (Schiedner et al. 2003).

### Barriers to spread of virus within the tumor

In the same manner as drug therapy, systemically administered virotherapy must cross the endothelial lining of blood vessels to enter the tumor interstitium, travel through extracellular matrix (ECM), basement membrane and necrotic areas, to access tumor cells (Molema et al. 1997). Tumor vasculature is disordered, with wide fenestrae and discontinuous basement membranes (Jain, 1987). Tumor size and connective tissue, however, pose significant impediments to viral spread through the tumor (Li et al. 2004). Oncolytic viruses are larger than conventional drugs, varying from 20–300 nm (Fisher, 2006). The diffusion of larger viruses, such as HSV, are significantly restricted by the ECM (McKee et al. 2006). The tumor microenvironment is highly heterogenous, with areas of hypoxia and acidosis, which can affect the virus target cell interaction (Vaha-Koskela et al. 2007). The pattern of tumor infection by oncolytic viruses, whether delivered intratumorally or systemically is heterogenous (Sauthoff et al. 2003; Kim et al. 2006; Shayakhmetov et al. 2002). For example although high levels of adenovirus persist in xenograft tumor tissue 8 weeks after intratumoral injection, distribution is patchy (Sauthoff et al. 2003).

Strategies designed to enhance the delivery of drugs to tumors (Minchinton and Tannock, 2006) can be applied to oncolytic viruses. Treatment of the tumor ECM by proteases could enhance virus spread (Kim et al. 2006; Kuriyama et al. 2000). An ECM degredation protein relaxin, expressed by an engineered adenovirus vector, increased tumor penetration by the virus and inhibited tumor growth in a xenograft model (Kim et al. 2006). Fusogenic membrane glycoproteins (FMG) are proteins involved in the entry of several types of virus, including measles and VSV into cells. FMGs induce widespread cell-to-cell fusion and the formation of giant syncitia (Bateman et al. 2000), which may enhance the ability of a virus to overcome the intratumoral barriers to spread (Ahmed et al. 2003).

## Conclusion

Oncolytic viruses are tumor selective potent anti-cancer agents. Currently in the early stages of their emergence as useful therapy, their promise is yet to be fulfilled. Clincial studies so far have provided evidence of limited activity both following local and systemic administration. Human tumors are very diverse, and the response to virotherapy is variable. Significant challenges remain to be overcome before oncolytic viruses can emerge as broadly useful therapy. Efforts are being made to enhance the anti-tumor potency of oncolytic viruses, whilst maintaining tumor selectivity. The anti-viral immune response is a major obstacle to effective virotherapy, although the release of TAAs and provision of a ‘danger’ signal offer the tantalising possibility of generating anti-tumor immunity. Rapid clearance of virus from the bloodstream has limited systemic application. A host of different solutions, including cellular carriers and modification of virus particles, have recently emerged to enhance systemic delivery. Oncolytic viruses have a favourable toxicity record, and safety concerns regarding the use of live replicating viruses continue to be addressed. A lack of cross-resistance, non-overlapping toxicity, and evidence of synergistic interactions with radiotherapy and chemotherapy lays the basis for future combination virotherapy treatment. The considerable concerns by institutions worldwide as to the toxicity of these agents to patients, staff, patients’ immediate relatives and shedding into the environment have resulted in more defined regulatory guidelines and appropriate risk assessments. This process combined with the considerable costs when compared to standard cancer chemotherapeutics has led to questioning of the viability of virus based approaches. Despite this scrutiny, it is clear that oncolytic viruses are moving closer to fulfilling their exciting clinical potential and may become the standard of care for certain cancer scenarios in the future.
